# Modelling the optimal dosing schedule for artemether-lumefantrine chemoprophylaxis against malaria

**DOI:** 10.1186/s13104-022-06212-y

**Published:** 2022-10-02

**Authors:** Joel Tarning, Lorenz von Seidlein, Arjen M. Dondorp, Nicholas J. White, Richard J. Maude

**Affiliations:** 1 Mahidol Oxford Tropical Medicine Research Unit, Faculty of Tropical Medicine, Mahidol University, Bangkok, Thailand; 2 Centre for Tropical Medicine and Global Health, Nuffield Department of Medicine, University of Oxford, Oxford, UK; 3 Harvard TH Chan School of Public Health, Harvard University, Boston, USA; 4 The Open University, Milton Keynes, UK

**Keywords:** modelling, artemether, lumefantrine, prophylaxis, malaria, pharmacometric

## Abstract

**Objective:**

Antimalarial chemoprophylaxis for high risk groups in endemic areas of Southeast Asia has the potential to reduce malaria transmission and accelerate elimination. However, the optimal choice of medication and dosing for many potential candidates is not clear. For a planned randomised controlled trial of prophylaxis for forest goers in Cambodia, artemether-lumefantrine (AL) was selected because of its ongoing efficacy and excellent tolerability and safety. As AL had not been used before for this purpose, a previously published pooled pharmacometric meta-model was used to determine the optimal dosing schedule.

**Results:**

A full 3 day AL treatment course given twice a month, and twice daily treatment given once a week, resulted in trough concentrations consistently above the therapeutic threshold of 200 ng/mL. However, the most favourable exposure profile, and arguably most practical dosing scenario, was an initial 3 day full AL treatment course followed by twice daily dosing given once a week for the duration of chemoprevention. The latter was adopted as the dosing schedule for the trial.

## Introduction

Antimalarial chemoprophylaxis for high risk groups in endemic areas of Southeast Asia has the potential to reduce malaria transmission and accelerate elimination. However, the optimal choice of medication and dosing for many potential candidates is not clear. In the Greater Mekong Subregion (GMS) of Southeast Asia, the high prevalence of artemisinin and multidrug resistant *P. falciparum* limits the choice of drugs suitable for chemoprophylaxis. Artemether-lumefantrine (AL) is one of the six WHO-recommended artemisinin-based combination therapies (ACTs) for treatment of uncomplicated *falciparum* and *vivax* malaria, but is not widely used for prophylactic purposes, because of the relatively short terminal elimination half-life, requiring a more frequent dosing [1]. However, because of the limited options, AL was selected as the preferred candidate drug combination for a randomised controlled trial of prophylaxis for forest goers in Cambodia [2] as it continues to be highly efficacious in the GMS for the treatment of *falciparum* and *vivax* malaria. AL is globally the most widely used antimalarial. It has excellent tolerability and safety profiles. Therapeutic success has been assessed in the treatment of acute uncomplicated *falciparum* malaria, but the aim of prophylactic treatment is to eliminate novel asymptomatic infections. Symptomatic patients with malaria have high levels of parasitaemia at treatment initiation, and the total biomass associated with preventing novel infections emerging from the liver is several orders of magnitude lower. There is a risk that targeting drug concentrations required for the treatment of symptomatic infections results in over-dosing when used in chemoprophylaxis. As AL has not been used previously for prophylaxis, a pharmacometric modelling and simulation approach was used to determine the optimal dosing schedule.

**Fig. 1:**
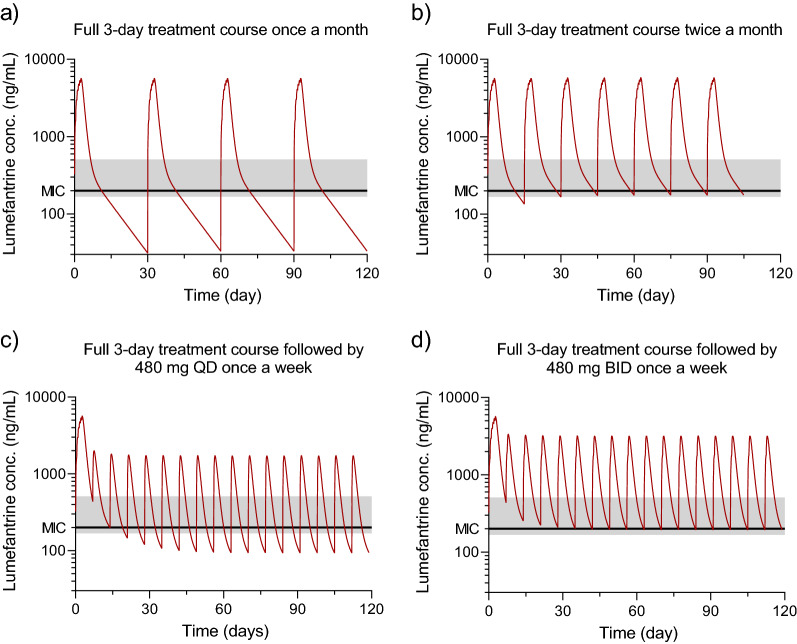
Predicted lumefantrine concentrations simulating different dosing schedules. Panel a) shows a full 3 day treatment course of 480 mg lumefantrine given once a month. Panel b) shows a full 3 day treatment course of 480 mg lumefantrine given twice a month. Panel c) shows a loading dose of a full 3 day treatment course of 480 mg lumefantrine followed by 480 mg lumefantrine QD given once a week. Panel d) shows a loading dose of a full 3 day treatment course of 480 mg lumefantrine followed by 480 mg lumefantrine BID given once a week

## Main text

Intermittent preventive treatment of malaria in high risk groups in endemic areas aims to maintain effective antimalarial drug concentrations in the body throughout the period of transmission exposure to eliminate newly acquired infections. In chemoprophylaxis with intermittent AL, the main drug effect is provided by the more slowly eliminated lumefantrine. Lumefantrine has a relatively short terminal elimination half-life of 3–5 days compared to many of the other partner drugs used in ACTs [1], and thus requires more frequent dosing to maintain effective concentrations.

A pharmacometric approach was used to evaluate different dosing regimens of lumefantrine, when used in chemoprophylaxis. Reported pharmacokinetic studies in healthy volunteers are often small and conducted in a homogenous population of young adult males, and parameter estimates, particularly between-subject variability associated with these parameters, are not generalizable to a wider population or to other geographical regions. Thus, a previously published pooled pharmacometric meta-model was used to simulate mean concentration–time profiles of lumefantrine, associated with different dosing [1]. This model is, to date, the largest pharmacometric model of lumefantrine, including 26 individual studies and close to 4000 patients. This should be the most reliable source of pharmacokinetic parameter estimates available when simulating lumefantrine exposures associated with novel dosing regimens.

The pharmacokinetic model consisted of a two-compartment distribution model, with a first-order absorption. Lumefantrine has dose-limited absorption resulting in a less than proportional increase in exposure with increasing dosing. Baseline parasitemia was also a covariate in the model, resulting in a lower exposure with increasing severity. To be conservative, and not over-estimate exposure associated with lower level of severity, we assumed that simulated individuals were similar to the typical patient in the original model (i.e. admission parasitemia of 15,800 parasites/μL). Uninfected individuals would have a higher exposure to lumefantrine and therefore an expected greater chemoprophylactic effect. Simulated individuals were assumed to be non-pregnant adults weighing 70 kg. A total of four dosing scenarios were evaluated (Fig. 1); (a) a full treatment course of 480 mg lumefantrine twice a day (BID) for 3 days, given once a month, (b) a full treatment course of 480 mg lumefantrine BID for 3 days, given twice a month, (c) a loading dose of a full treatment course of 480 mg lumefantrine BID for 3 days followed by 480 mg lumefantrine QD given once a week, and (d) a loading dose of a full treatment course of 480 mg lumefantrine BID for 3 days followed by 480 mg lumefantrine BID given once a week. All simulations were performed in the software Berkley Madonna. The simulated concentration–time profiles illustrate a typical exposure profile, overlaid with reported day 7 concentrations of lumefantrine associated with therapeutic success. The therapeutic day 7 lumefantrine concentrations published to date range from 170 ng/ml to 500 ng/mL [3–9]. A pooled analysis including a total of 2787 patients reported that day 7 concentrations ≥ 200 ng/mL were associated with > 98% 28 day cure rates in treatment studies [10].

A full 3 day treatment course given twice a month, and twice daily treatment given once a week, resulted in trough concentrations consistently above the therapeutic threshold of 200 ng/mL. However, the most favourable exposure profile, and arguably most practical dosing scenario was an initial 3 day full treatment course followed by twice daily dosing given once a week for the duration of chemoprevention.

## Limitations

The model was developed to simulate dosing in people with malaria, but volunteers were excluded if they had clinical malaria at baseline. Patients with malaria are expected to have lower drug exposure compared to uninfected individuals, suggesting that model-based assumptions will not lead to under-dosing when used as prophylactic treatment. It was deemed more appropriate to use a pharmacokinetic model with reliable parameter estimates in patients, compared to a small trial published in healthy volunteers, since the only drawback would be a possible under-estimation of drug exposure and expected prophylactic efficacy.

The cut-off for therapeutic success used here was based on the treatment of acute uncomplicated *falciparum* malaria, but the aim of prophylactic treatment is to eliminate novel asymptomatic infections. Symptomatic patients with acute uncomplicated malaria have high levels of parasitaemia (10^9^–10^11^ parasites) at treatment initiation. However, the total biomass associated with novel infections emerging from the liver is several orders of magnitude lower (10^4^–10^5^ parasites). There is a risk that the therapeutic cut-off chosen here results in a degree of over-dosing, and that once weekly QD treatments might also be effective in preventing malaria. However, therapy with AL is safe and well-tolerated and lower doses carry a higher risk of resistance development and should be avoided.

The model was based on studies conducted in both Africa and Asia, including one in Cambodia. There may be differences in dosing between ethnicities although these have not been demonstrated to date.

Finally, AL should be administered with a small amount of fat to maximise the absorption of lumefantrine, and this is relatively easy to control in a treatment setting. These recommendations might not always be followed and could therefore result in reduced absorption and lower drug concentrations of lumefantrine.

## Data Availability

Not applicable.

## Abbreviations

ACT: Artemisinin-based combination therapy
AL: Artemether lumefantrine
BID: Twice a day
QD: Once daily
